# Supplementary material for: Altered microRNA Transcriptome in Cultured Human Liver Cells upon Infection with Ebola Virus
Source: Int J Mol Sci. 2021 Apr 6;22(7):3792. doi: 10.3390/ijms22073792 (PMC8038836; doi:10.3390/ijms22073792)
Supplement: Supplementary file 1 [file ijms-22-03792-s001.zip › Supplementary_File/C_ GO_Analysis_Results/16-30nt_go_Makona-96h-Huh7_vs_Control-96h-Huh7_up.mature_mirna_targets/CC_Pvalue_tree.pdf]

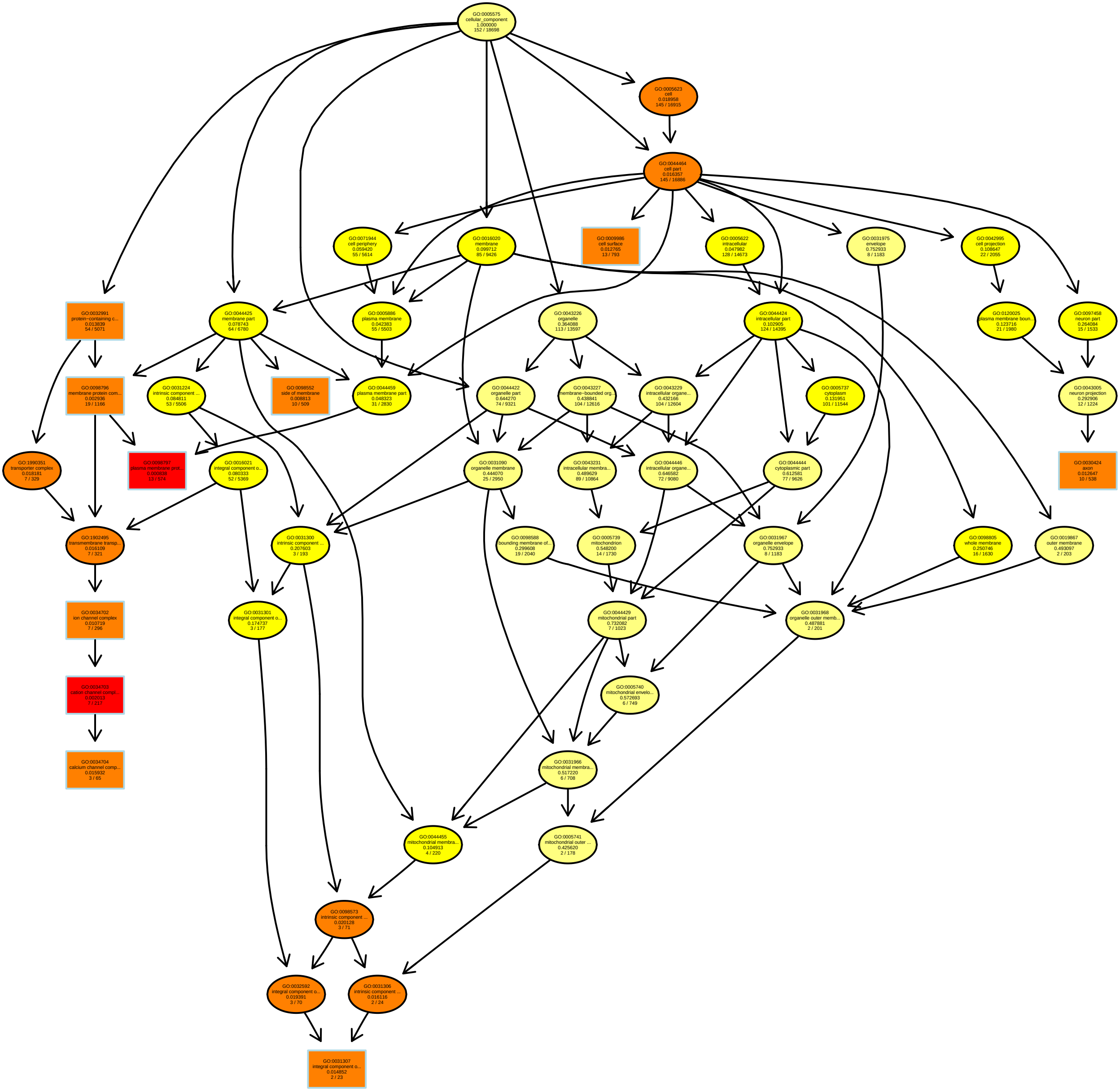

GO:0005575 cellular_component 1.000000 152 / 18698
GO:0005623 cell 0.018958 145 / 16915
GO:0044464 cell part 0.016357 145 / 16886
GO:0071944 cell periphery 0.059420 55 / 5614
GO:0016020 membrane 0.099712 85 / 9426
GO:0009986 cell surface 0.012765 13 / 793
GO:0005622 intracellular 0.047982 128 / 14673
GO:0031975 envelope 0.752933 8 / 1183
GO:0042995 cell projection 0.108647 22 / 2055
GO:0032991 protein−containing c... 0.013839 54 / 5071
GO:0044425 membrane part 0.078743 64 / 6780
GO:0005886 plasma membrane 0.042383 55 / 5503
GO:0043226 organelle 0.364088 113 / 13597
GO:0044424 intracellular part 0.102905 124 / 14395
GO:0120025 plasma membrane boun... 0.123716 21 / 1980
GO:0097458 neuron part 0.264084 15 / 1533
GO:0098796 membrane protein com... 0.002936 19 / 1166
GO:0031224 intrinsic component ... 0.084811 53 / 5506
GO:0098552 side of membrane 0.008813 10 / 509
GO:0044459 plasma membrane part 0.048323 31 / 2830
GO:0044422 organelle part 0.644270 74 / 9321
GO:0043227 membrane−bounded org... 0.438841 104 / 12616
GO:0043229 intracellular organe... 0.432166 104 / 12604
GO:0005737 cytoplasm 0.131951 101 / 11544
GO:0043005 neuron projection 0.292906 12 / 1224
GO:1990351 transporter complex 0.018181 7 / 329
GO:0098797 plasma membrane prot... 0.000838 13 / 574
GO:0016021 integral component o... 0.080333 52 / 5369
GO:0031090 organelle membrane 0.444070 25 / 2950
GO:0043231 intracellular membra... 0.489629 89 / 10864
GO:0044446 intracellular organe... 0.646582 72 / 9080
GO:0044444 cytoplasmic part 0.612581 77 / 9626
GO:0030424 axon 0.012647 10 / 538
GO:1902495 transmembrane transp... 0.016109 7 / 321
GO:0031300 intrinsic component ... 0.207603 3 / 193
GO:0098588 bounding membrane of... 0.299608 19 / 2040
GO:0005739 mitochondrion 0.548200 14 / 1730
GO:0031967 organelle envelope 0.752933 8 / 1183
GO:0098805 whole membrane 0.250746 16 / 1630
GO:0019867 outer membrane 0.493097 2 / 203
GO:0034702 ion channel complex 0.010719 7 / 296
GO:0031301 integral component o... 0.174737 3 / 177
GO:0044429 mitochondrial part 0.732082 7 / 1023
GO:0031968 organelle outer memb... 0.487881 2 / 201
GO:0034703 cation channel compl... 0.002013 7 / 217
GO:0005740 mitochondrial envelo... 0.572693 6 / 749
GO:0034704 calcium channel comp... 0.015932 3 / 65
GO:0031966 mitochondrial membra... 0.517220 6 / 708
GO:0044455 mitochondrial membra... 0.104913 4 / 220
GO:0005741 mitochondrial outer ... 0.425620 2 / 178
GO:0098573 intrinsic component ... 0.020128 3 / 71
GO:0032592 integral component o... 0.019391 3 / 70
GO:0031306 intrinsic component ... 0.016116 2 / 24
GO:0031307 integral component o... 0.014852 2 / 23
